# Do You Get What I Mean?!? The Undesirable Outcomes of (Ab)Using Paralinguistic Cues in Computer-Mediated Communication

**DOI:** 10.3389/fpsyg.2021.658844

**Published:** 2021-05-12

**Authors:** Yael Sidi, Ella Glikson, Arik Cheshin

**Affiliations:** ^1^Department of Education and Psychology, The Open University of Israel, Ra’anana, Israel; ^2^Graduate School of Business Administration, Bar Ilan University, Ramat Gan, Israel; ^3^Department of Human Services, University of Haifa, Haifa, Israel

**Keywords:** computer-mediated communication, paralinguistic cues, social information processing theory, impression management, self-presentation

## Abstract

The shift to working from home, which has intensified due to Covid-19, increased our reliance on communication technology and the need to communicate effectively via computer-mediated communication and especially via text. Paralinguistic cues, such as repeated punctuation, are used to compensate for the lack of non-verbal cues in text-based formats. However, it is unclear whether these cues indeed bridge the potential gap between the writer’s intentions and the reader’s interpretations. A pilot study and two experiments investigated the effect of using repeated punctuation on behavioral intention to assist an email writer in a work-related situation. Findings demonstrate that while the intentions behind using repeated punctuation relate to signaling situational importance or affective state, behavioral intentions are driven by dispositional rather than situational attributions. Specifically, the use of repeated punctuation reduces perceived competence of the message writer and consequently decreases positive behavioral intentions. Overall, the study challenges the simplified view of paralinguistic cues as communication facilitators, highlighting their potential harmful effects on impression formation and behavioral intentions in the digital age.

## Introduction

Digital technology enables people to work away from the office, at least part of the time, and this shift has intensified due to the Covid-19 pandemic (Bick et al., 2020; Kramer and Kramer, 2020; Nguyen et al., 2020; Ohme et al., 2020), expanding to a new extreme the reliance on communication technology and the need to communicate effectively via computer-mediated communication (CMC). Specifically, more and more communication is being conveyed via text-based media, such as emails (Radicati and Levenstein, 2015; Goodman-Deane et al., 2016), known to be lean in social cues compared to face-to-face (FTF), video, or voice interactions (Daft and Lengel, 1986). While emails enable quick communication and increase productivity (Derks and Bakker, 2010), people often experience work-related email overload (Dabbish and Kraut, 2006), which is no surprise as an enormous amount of work emails is being exchanged daily (app. 130 billion according to Radicati and Levenstein, 2015). Clearly, not all emails are equally important or urgent to the email writer. Cues, such as repeated punctuation (“!!” and “??”), are often used as non-verbal signaling of importance (Oberlander and Gill, 2006). Adding various cues to emails is prevalent (Kalman and Gergle, 2014); however, their impact on message interpretation has received scant attention. When on the receiving end, do we interpret the use of repeated punctuation as a sign of importance, and do we respond accordingly? The present study’s aim is to systematically investigate how the use of repeated punctuation shapes the interpretation and consequent behavioral intentions of the message receiver in work-related email communication.

Email usage as well as other text-based communication forms is expected to continuously grow in coming years, as it warrants sufficient control over the information being shared and the way the message is crafted, allowing the writer to actively influence self-representation, even more so than in FTF interaction or videoconferencing (Wells and Dennis, 2016; Marlow et al., 2017). However, as emails are asynchronous and relatively lean in social information, they leave much room for the reader’s interpretation. Due to this interpretation challenge, readers tend to actively search for additional information embedded within an email text that can help with the interpretation process, including paralinguistic cues (Byron, 2008; Johri, 2012; Tong and Walther, 2015). Moreover, the growing reliance on text-based digital services (Przegalinska et al., 2019; Yang, 2021) has led to the use of programmed chat bots. It is important that these digital tools “understand” the true intention of customers and, as importantly, communicate back in such a manner that would lead to the clearest and most effective message. Thus, understanding the impact these cues have on text-based interaction and communication is paramount.

Paralinguistic cues in written texts, such as repeated punctuation, are defined as written communicational codes (e.g., words and symbols) which supplement written language and signify a socially shared gist (Lea and Spears, 1992; Luangrath et al., 2017). Social information processing theory suggests that paralinguistic cues are utilized in place of non-verbal cues to overcome CMC limitations of lack of social presence (Walther, 2007, 2016). Overall, paralinguistic cues have been argued to enrich the expressiveness of the text, disambiguate it, convey affect and a sense of immediacy, and emphasize intended statements (Aldunate and González-Ibáñez, 2017; Prada et al., 2018; Liu and Sun, 2020). However, some empirical research that examined interpretation of paralinguistic cues in textual communication has challenged this notion. For example, Riordan and Trichtinger (2017) found that paralinguistic cues did not facilitate accurate interpretations of email messages, despite feelings of (over)confidence in perceiving the intended message. Thus, whether paralinguistic cues are accurately interpreted and convey the communicator’s intentions remains an open question (Scott and Fullwood, 2020).

Repeated punctuation marks serve as *expressive cues*, to communicate affect and clarify context and meaning (Gill and Oberlander, 2002; Oberlander and Gill, 2006). If perceived as such, emails containing repeated punctuation should convey and be interpreted as an indication of high importance or urgency. Yet, we know that people do assess not only a message but also the messenger. There is a plethora of research showing how people interpret and attribute situational as well as dispositional inferences when judging actions of others (e.g., Cramton et al., 2007; Kammrath et al., 2007; van Kleef et al., 2012; Hareli, 2014; Elsbach and Bechky, 2017; Riordan and Glikson, 2020). Two fundamental dimensions on which people predominantly judge others are warmth and competence (Judd et al., 2005; Fiske et al., 2007). *Warmth* reflects perceived social intentions (e.g., friendliness), while *competence* stands for perceived capacity to achieve goals (e.g., skill). Notably, these judgments were found to beget important behavioral intentions in social settings, such as deciding whether or not to hire a candidate for a job (Cuddy et al., 2011). Thus, it is not merely interpretations; these interpretations have consequences on future actions.

While email norms are still evolving and change between groups and teams (Cheshin et al., 2013; Glikson and Erez, 2013), research suggests that formal and conservative use of accurate punctuation, spelling, and grammar is the expected etiquette (Pankoke-Babatz and Jeffrey, 2002; Lewin-Jones and Mason, 2014). Politeness (e.g., correct grammar) increases positive views of the message and of the sender (Jessmer and Anderson, 2001), while etiquette violations negatively shape readers’ perceptions of email writers (Vignovic and Thompson, 2010). Thus, repeated punctuation might violate expression norms or might be deemed as inappropriate, thereby leading to negatively biased dispositional attributions and responses. For example, Vareberg and Westerman (2020) found that for first impression of instructors, using paralinguistic cues affected their perceptions by students such that they were perceived to be more caring, yet less competent. Namely, paralinguistic cues are not always helpful and in some cases might even harm readers’ perceptions of the writer, creating a gap between the writer’s intentions and the reader’s interpretation that shapes the reader’s response.

In the present paper, we aimed to test whether repeated punctuation increases perceived message importance and thus facilitates positive behavioral intentions, or whether they have a negative impact on dispositional attributions thus hindering positive behavioral intentions. Therefore, this work puts into test two competing hypotheses:

*(H1a) Work-related email containing repeated punctuation marks will lead to higher perceptions of situational attribution (e.g., perceived importance) compared to email with one punctuation mark, and the situational attribution will mediate the relationship between punctuation marks and positive intentional behaviors toward the email writer.*

*(H1b) Work-related email containing repeated punctuation marks will lead to lower perceptions of dispositional attribution* (e.g., *competence and warmth) compared to email with one punctuation mark, and the dispositional attributions will mediate the relationship between punctuation marks and positive intentional behaviors toward the email writer.*

## Pilot—Repeated Punctuation Intention Survey

The underlying assumption of the present study, based on CMC literature described above, is that people use repeated punctuation in CMC to convey situational cues. Purposely, they wish to convey through these cues the importance or immediacy of their message and express their emotions and are not intentionally attempting to convey information about their character. To establish this assumption, we conducted a pilot study. Sixty-three undergraduate students from a large American university (52% female, *M*_*age*_ = 25.9) filled the questionnaire on a voluntarily basis. The questionnaire described the use of multiple question marks in CMC and consisted of a variety of filler questions regarding paralinguistic habitual use. The relevant question for the present work regarded the role of repeated question marks and, particularly, what they convey. Items included multiple affective reasoning (“negative affective state”) and contextual reasoning (e.g., “emphasizing the urgency of the issue”), as well as person-related reasoning (e.g., “inability of the person to express themselves with words”) rated on a Likert scale from 1 (“*Strongly disagree*”) to 7 (“*Strongly agree*”). As expected, the affective and contextual reasons received significantly higher ratings (*M* = 4.32, SD = 1.22, *Cohen’s d* = 0.56, *M* = 4.63, SD = 1.57, *Cohen’s d* = 0.68, respectively) than person-related reasons (*M* = 3.58, SD = 1.54), *p’s* ≤ 0.006. Moreover, only affective and contextual reasons differed significantly from the scale’s medium value (*p’s* < 0.001). These results offer empirical support to the theoretical notion that paralinguistic cues in CMC are perceived as a means to convey information about the context the writer is experiencing (i.e., situational), rather than information about the writer’s traits or personality (i.e., dispositional).

## Experiment 1

We examined the effect of using repeated question marks on situational and dispositional interpretation of an email message and consequent behavioral intentions. A scenario relatable to our sample population was selected (a question regarding employment opportunity of a student). Notably, past research has shown that the use of paralinguistic cues in CMC is gender biased and is associated more with women (Wolf, 2000; Glikson et al., 2018). For instance, Marlow et al. (2017) found that using a salutation with an exclamation mark harmed the perception of competence for female, but not for male writers. Butterworth et al. (2019) found that perceptions of appropriateness and likability of email writers were also malleable to gender and type of emoji used. Thus, the use of repeated punctuation by women might strengthen negative female stereotypes, which are associated with over-expressiveness and are generally related to negative trait assessments (Greenwood and Isbell, 2002; Barrett and Bliss-Moreau, 2009). We therefore examined gender as a potential moderator:

*(H3) Writer’s gender will moderate the relationship between repeated punctuation marks and email interpretation (situational or dispositional), and consequently the effect of repeated punctuation marks on behavioral intentions. Specifically, the relationships will be weaker for males compared to females.*

### Method

#### Participants

To determine sample size, we used a power analysis (using GPower 3.1) for a linear regression with four predictors. We assumed a medium effect size (*f*^2^ = 0.15), and based on an α = 0.05 and power = 0.95, the desired sample size was 129. One hundred and forty-two Dutch undergraduates participated in the experiment for course credit (58% females; *M*_*age*_ = 21.1).

#### Materials and Procedure

Participants received a link to an online survey, which included a task description for evaluating an applicant for a position in the university. They were asked to assist with the selection procedure for the position by expressing their impression of one of the applicants, which was randomly selected for their review. In practice, all participants reviewed the same “applicant.” Participants were presented with an English email written by the applicant (see Appendix), including basic information about the applicant (e.g., previous experience with a similar position, age) and two questions (e.g., “Is this position still relevant?/???” and “How soon does the project begin?/???”), in one of four possible conditions, randomly assigned. In all the conditions, the emails were identical except for the number of question marks used (one or three) and the gender of the applicant, signified by a male or female name. Participants rated the applicant and their willingness to recommend the applicant for the position.

#### Measures

Trait attributions were comprised of warmth and competency, measured by an adapted scale (Cuddy et al., 2007), and included the following items for warmth (Cronbach’s α = 0.80): “nice,” “positive,” and “friendly” and for competence (α = 0.83): “competent,” “intelligent,” and “professional.” Importance of the position reflected situational attributions and was measured explicitly using three items (α = 0.88; e.g., “*This position is important to the applicant*”). Willingness to recommend was measured explicitly using four items (α = 0.93; e.g., “*I think that this applicant fits this position*”). All measures were assessed using seven-item Likert-like scales (1 = not at all” and 7 = “to a great extent”).

### Results and Discussion

**FIGURE 1:**
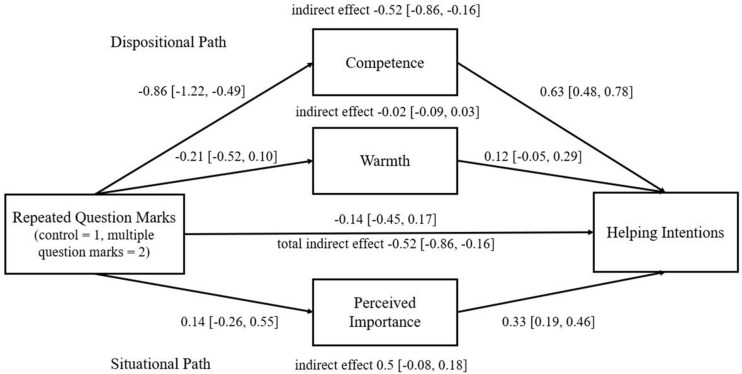
Experiment 1 multiple-mediation model of the effect of use of repeated question marks (vs. one question mark) on helping intentions. *N* = 142; beta coefficients and confidence intervals are presented based on PROCESS Model 4 (Hayes and Preacher, 2013), controlling for writer’s gender. Overall model—*F*(5,136) = 46.08, *R*^2^ = 0.63 (*R*^2^*med* = 0.42—explained variance of the mediation is calculated based on Fairchild and MacKinnon, 2009).

Means, standard deviations, and correlations are summarized in Table 1. To test the indirect effect of multiple question marks on behavioral intentions, we used PROCESS Model 4 for SPSS 25 (Hayes and Preacher, 2013; see Bass et al., 2019 and Yu et al., 2019 for a similar procedure), where the model included warmth and competence (dispositional interpretation) as well as the perceived importance of inquiry (situational attribution) as mediators and the applicant gender as a covariate. A total indirect effect was found significant (bootstrap sample 10,000; 95 CI%: [−0.86, −0.16]), yet, when looking at each indirect effect separately, it revealed that only competence mediated the relationship (*competence* indirect effect [−0.86, −0.16], *warmth* indirect effect [−0.09, 0.03], and *perceived importance* indirect effect [−0.08, 0.18], see Figure 1). Thus, only competence was impacted by the repeated question marks and mediated the relationship to the positive behavioral intention of recommending the applicant.

**TABLE 1:** Descriptive statistic and correlations for Experiment 1.

	**Mean**	**SD**	**(1)**	**(2)**	**(3)**	**(4)**	**(5)**	**(6)**	**(7)**
(1) Repeated punctuation marks^a^	1.43								
(2) Writer’s gender^b^	1.53	0.5	0.14						
(3) Perceived writer competence	3.71	1.17	−0.36*	0.01					
(4) Perceived writer warmth	4.57	0.91	–0.11	–0.05	0.44*				
(5) Perceived message importance inquiry	4.47	1.19	0.06	0.02	0.43*	0.34*			
(6) Positive behavioral intentions	3.54	1.32	−0.25*	–0.07	0.74*	0.43*	0.56*		
(7) Age	21.12	3.35	0.07	–0.08	0.03	0.06	0.02	0.01	
(8) Participant’s gender	1.58	0.49	0.02	0.02	–0.01	–0.02	0.01	0.01	–0.09

^*a*^−1 = no repeated punctuation. 2 = repeated punctuation; ^*b*^−1 = male, 2 = female. *p < 0.05.

• Repeated question marks—the manipulation of number of repeated question marks (?/???)
• Competence—perception of the writer as being competent (dispositional attribution)
• Warmth—perception of the writer as being warm and friendly (situational attribution)
• Perceived importance—perception of the issue as being important and urgent (situational attribution)
• Helping intentions—the willingness to recommend the applicant.

To test the possible moderation of the writer’s gender, we used PROCESS Model 8 (Hayes and Preacher, 2013), yet no moderation was found (bootstrap sample 10,000; 95 CI% index of moderated mediation *competence* [−0.665, 0.298]; *warmth* [−0.222, 0.041]; *importance* [−0.192, 0.371]). (Moderated mediation induced by PROCESS Model 7 was also insignificant.) Thus, gender had no impact on this relationship.

In sum, our findings provide initial evidence for the adverse effect of using repeated punctuation in an email on perceptions of competence and behavioral intentions toward the writer. We did not find the expected moderating effect of gender, suggesting that the use of repeated question marks hindered perceptions regardless of the writer’s gender.

## Experiment 2

In Experiment 2, we sought to look at the external validity of our findings by examining other types of repeated punctuations (e.g., “!!” and “?!”) in more ecologically valid stimuli. Thus, we collected “real-life” work-related email correspondences containing paralinguistic cues and presented them as the stimuli in our experiment. To further understand the process by which people interpret paralinguistic cues, we aimed to examine whether the clarity of the message content would affect the relationship between using repeated punctuation and interpretation. As mentioned, paralinguistic cues are often used to clarify the message and its context (Houghton et al., 2018). Therefore, the use of paralinguistic cues with ambiguous messages should be more justifiable than such use when the content of the message is clear. This means that for ambiguous messages the use of repeated punctuation could be less harmful with regard to dispositional attributions and intentional behaviors than such use for messages with a clear content. This novel factor was examined in Experiment 2 by asking participants to rate the clarity of the message. As gender did not moderate our findings in Experiment 1, we did not address it in Experiment 2.

*(H4) Message clarity will moderate the relationship between repeated punctuation marks and perceptions of competence, and consequently the effect of repeated punctuation marks on behavioral intentions. Specifically, the relationships will be stronger for clear messages compared to more ambiguous messages.*

### Method

#### Participants

To determine sample size, we used a power analysis (using GPower 3.1) for a linear regression with five predictors. We assumed a small effect size (*f*^2^ = 0.15), and based on an α = 0.05 and power = 0.95, the desired sample size was 107. One hundred Israeli MBA students participated in the experiment for course credit (51% females; *M*_*age*_ = 30.58); however, due to missing data for six participants, the final sample was 94.

#### Materials and Procedure

Prior to the experiment, as part of a different project, graduate students at a public university, who were engaged in a part-time program and were working outside the university, shared their recent work-related emails (in Hebrew). Prior to sharing the correspondences, all identifying details and sensitive information were deleted. Emails were categorized based on their length, paralinguistic cues, and content. Three short independent emails with repeated punctuation marks (e.g., “??,” “!!,” and “?!”) were chosen. In order to create the paralinguistic cue manipulation, researchers removed the repeated punctuation marks from the original messages, creating two conditions: original emails with repeated punctuation marks and manipulated emails for which repeated punctuation marks were removed. All other email features were identical (see Appendix).

Participants received a link to the survey, which included a task description for evaluating work email messages. They were randomly assigned to one of the two conditions—original messages with repeated punctuation marks or fixed messages with no repeated punctuation marks. In all conditions, three independent emails were presented; they were identical except for the number of punctuation marks used. Following the presentation of each email, participants rated their impressions and interpretations.

#### Measures

The same trait measure from Experiment 1 was used for competency (α = 0.88). As we did not find any effect for warmth in Experiment 1, we instead examined a different situational attribution—perceived writer’s negative affective state. The measure, adapted from the PANAS scale (Watson et al., 1988), comprised two items (*“The message conveys negative mood,” “While writing the message the author was in a negative mood*,” α = 0.90). Importance of the email reflected situational attributions and was measured using two items (“*This message is important to the email writer*” and “*This message is urgent to the email writer*,” α = 0.88). Willingness to assist was measured explicitly using four items *(“I would do anything to help the email writer,”* “*I would give this email priority*,” *“I would put aside other things to handle this email,”* and *“I would extend special efforts to address this email*,” α = 0.93). In addition, we asked participants to rate the clarity of the message *(“The message was clear”* and *“The message was unambiguous”*; α = 0.70). All measures were assessed for each email separately, using seven-item Likert-like scales (1 = “not at all” and 7 = “to a great extent”).

### Results and Discussion

**FIGURE 2:**
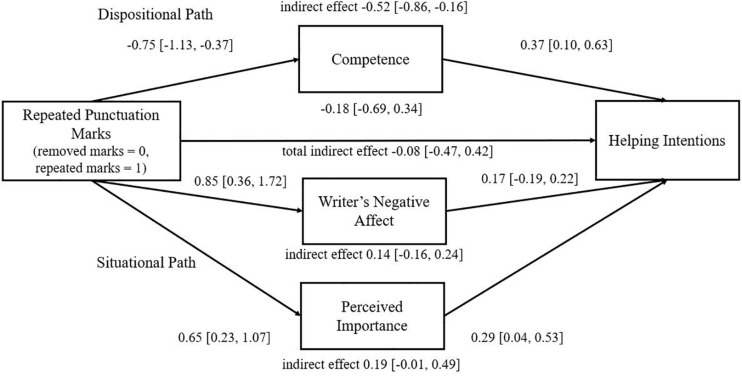
Experiment 2 multiple mediation model of the effect of use of repeated punctuation (vs. control) on helping intentions. *N* = 92; beta coefficients and confidence intervals are presented based on PROCESS Model 4 (Hayes and Preacher, 2013), controlling for perceived clarity of the message. [Overall model—*F*(5,88) = 4.83, *R*^2^ = 0.22; *R^2^med* = 0.05—explained variance of the mediation is calculated based on Fairchild and MacKinnon, 2009]

Means, standard deviations, and correlations are summarized in Table 2. To test the indirect effect of multiple paralinguistic cues on behavioral intentions, we used PROCESS Model 4, where the model included competence (trait attribution) as well as the perceived importance of inquiry and writer’s negative mood (situational attributions) as mediators, and message clarity as a covariate. The total indirect effect was insignificant (bootstrap sample 10,000; 95 CI%: [−0.47, 0.42]). However, when looking at each indirect effect separately, it revealed that similarly to Experiment 1, only competence significantly mediated the relationship between repeated punctuation and helping intentions (*competence* indirect effect [−0.54, −0.04], *perceived writer’s negative mood* indirect effect [−0.17, 0.24], *perceived importance* indirect effect [−0.01, 0.49], see Figure 2). When the model was run with competence as a mediator and other variables (writer’s negative mood, importance, and message clarity) as covariates, the indirect effect was significant—bootstrap sample 10,000; 95 CI%: [−0.56, −0.04]. Thus, the results indicate that only the trait of competence mediated the relationship between the use of repeated punctuation marks and behavioral intention.

**TABLE 2:** Descriptive statistic and correlations for Experiment 2.

	**Mean**	**SD**	**(1)**	**(2)**	**(3)**	**(4)**	**(5)**	**(6)**	**(7)**
(1) Repeated punctuation marks^a^	0.48	0.50							
(2) Perceived writer competence	2.78	1.02	−0.36**						
(3) Perceived message importance	4.79	1.10	0.28**	0.16					
(4). Writer’s negative affect	4.42	1.23	0.35**	−0.33**	0.35**				
(5) Message clarity	3.73	1.19	–0.08	0.20*	0.26*	–0.04			
(6) Positive behavioral intentions	3.63	1.16	–0.07	0.35**	0.25*	0.01	–0.04		
(7) Age	30.14	5.92	0.17	–0.01	0.07	–0.07	−0.22*	–0.06	
(8) Participant’s gender^b^	1.52	0.50	0.03	–0.16	0.14	0.06	0.10	0.02	−0.30**

^*a*^−0 = no repeated punctuation, 1 = repeated punctuation. ^*b*^−1 = male, 2 = female. *p < 0.05. **p < 0.01.

• Repeated punctuation marks—the manipulation of repeated punctuation marks (repeated marks/no repeated marks)
• Competence—perception of the writer as being competent (dispositional attribution)
• Writer’s negative affect—perception of the writer as experiencing negative affect (situational attribution)
• Perceived importance—perception of the issue as being important and urgent (situational attribution)
• Helping intentions—the willingness to give a priority to the message.

The moderation of message clarity was tested using PROCESS Model 7 (Hayes and Preacher, 2013). A significant index of moderated mediation for perceived competence was found (bootstrap sample 10,000; 95 CI% [−0.33, −0.01]; *F*(4,89) = 4.66, *R*^2^ = 0.17). A simple slope analysis (Aiken and West, 1991) revealed that for high (+1 SD) and average levels of message clarity, the impact of paralinguistic cues was significant, lowering the perceived competence (*b* = −1.28, SE = 0.26, *p* < 0.001; *b* = −0.75, SE = 0.18, *p* < 0.001, respectively), but when the message clarity was low, the impact of repeated punctuation was insignificant (*b* = −0.22, SE = 0.26, *p* = 0.41). Akin, the mediation effect of competence on helping behaviors was significant for average and high levels of message clarity, but not for low levels of message clarity. This means that helping behaviors were hindered by perceptions of low competence only when message clarity was relatively high.

## General Discussion

Previous research regarding the role of paralinguistic cues in CMC considered them as facilitators in text interpretation, especially when forming first impressions (McAndrew and De Jonge, 2011). Correspondingly, research has found that paralinguistic cues are utilized under the assumption that they will facilitate the understanding of writer’s emotional state or the current situation (Spears and Lea, 1994; Houghton et al., 2018). The pilot survey we conducted offers further support for these findings, indicating that people believe that paralinguistic cues, particularly repeated question marks, are intended to convey situational information rather than reflecting the writer’s traits. However, scarce research has examined the “other side,” namely, what attributions people make when encountering paralinguistic cues.

The present paper addresses this gap by examining a model of situational and dispositional attributions for repeated punctuation in work-related email messages. We used repeated punctuation marks, which are associated with expression of emotional states (Mwangi et al., 2014), but not with people’s personality. Our findings show that people make more trait (i.e., stable) attributions related to paralinguistic cues: In two experiments, the paralinguistic cues were interpreted as representative of the trait of the writer in a negative way, which was related to the reporting of worse outcomes to the (ab)user of those cues.

A potential explanation for this effect stems from applying the Fundamental Attribution Error (Ross, 1977; Berry and Frederickson, 2015) principle, exemplifying the relevance of social biases to CMC. Indeed, research has shown that text-based communication is prone to stereotypes and heuristic decision making (Walther, 1996; Johri, 2012). For example, Vignovic and Thompson (2010) demonstrated that readers form negative perceptions of the email writer when emails contained technical language violations. To the best of our knowledge, the present study is the first to demonstrate the powerful effect of social biases in interpretation of repeated punctuation for behavioral intentions. Future research should explore the relations between other paralinguistic cues and social biases in text-based communication.

Despite expectations (Wolf, 2000; Glikson et al., 2018), we found no evidence for gender moderation. This finding joins others who maintain that gender does not play a role when discussing paralinguistic cues and their effects (Fullwood et al., 2013). This gender-blind phenomenon can be attributed to the strength of communication norms, violation of which drives negative attributions for both genders. In contrast, we did find a moderating role of message clarity. When messages were perceived as ambiguous, repeated punctuation cues did not have an adverse effect. Only when the content of message was clear did these added cues have a negative impact. This highlights the role of message clarity as a novel potential moderator for interpretation of paralinguistic cues in CMC, suggesting that when people do not understand the written message, they try to use additional cues as information and do not penalize the punctuation (ab)user.

Our findings allow inferring about expected norms in text-based CMC, adding insight to this growing literary domain (Marlow et al., 2017). In contrast to Houghton et al. (2018) who demonstrated a negative effect of over-formality in text-messaging among acquaintances, we found a negative effect of informality in email correspondence among strangers. The use of these additional textual cues, which attempt to signal situational or emotional elements to the lean text message, might be assessed as an inappropriate emotional display. Thus, the negative consequences we find in our experiments might be related to interpretation of these cues as an inappropriate emotional display (Cheshin, 2020). Specifically, the consequential negative attributions for (ab)using repeated punctuation across our experiments imply that formality is expected in various types of email correspondence, when interacting with strangers, as previously found in academic situations between students and professors (Stephens et al., 2009). Future research should include these different findings to investigate the exact boundaries of each condition, and the role of communication media and mode, as well as different types of communicators’ relationships, in readers’ interpretation of text-based communications.

There are some limitations to this work. First, the interaction scenarios used in both experiments were one-sided and asynchronous, namely, we did not examine reciprocal or synchronous communications. While the focus of the present study was impression formation of novel situations, it will be interesting to further examine how reciprocal interactions play out when repeated paralinguistic cues are used. In addition, the effect of repeated paralinguistic cues could be influenced by the hierarchical roles of the correspondents. Future research should explore whether the unequal social roles between the writer and the reader moderate the repeated marks impact. Second, these were all lab-based studies; despite the use of real emails in Experiment 2, the evaluations given were requests from researchers, and we can only assume that these also take place in real-life settings. Third, we ran our experiments in two different countries with different cultural norms and values. The first experiment was run in the Netherlands, in English, while the second experiment was run in Israel in Hebrew. Since the findings are consistent, we have confidence that these findings are robust; however, future research should test the effect in additional cultures and languages where the use of paralinguistic cues has different grammatical rules, as well as in cross-cultural communication.

The results of this work offer important practical implications for communicating via text-based electronic means. People use paralinguistic cues in CMC assuming that they will facilitate message interpretation (e.g., Riordan and Kreuz, 2010). Our findings suggest that these cues should be used with caution. Particularly, when abused, they might communicate inferior cognitive skills and result in a decreased willingness to assist the writer. Uncovering the biases in text interpretation may be helpful for text data analyses and creation of automated assistance for people using text-based service but also reply services, such as chat bots. The ability of an automated machine not only to correctly interpret the use of paralinguistic cues but also to implement their meaning in their own communication could be highly valuable for their effectiveness.

In conclusion, the lean text medium, which is becoming more and more prominent in our communication life, includes a paradox. Due to its leanness in non-verbal cues, writers add various paralinguistic cues to bring the text more to life, highlighting elements and adding more intonation and affect to the text. Yet, this attempt is futile, at least when it comes to (ab)using repeated punctuation such as question marks and exclamation points—and it may even lead to adverse consequences. Repeated punctuation did not lead to situational interpretation but rather to stable dispositional ones. When it comes to text-based communication, the gap between the intention of the writer and the interpretation should be minded.

## Data Availability Statement

The raw data supporting the conclusions of this article will be made available by the authors, without undue reservation.

## Ethics Statement

The studies involving human participants were reviewed and approved by Mark Rotteveel, University of Amsterdam and Nira Munichor, Bar Ilan University. The patients/participants provided their written informed consent to participate in this study.

## Author Contributions

All authors have contributed equally to this work. YS, EG, and AC equally contributed to the conception and design of the study and wrote sections of the manuscript. EG and AC performed the experiments. EG performed the statistical analyses. AC created the figures. YS wrote the first draft of the manuscript. All authors contributed to manuscript revision, read, and approved the submitted version.

## Conflict of Interest

The authors declare that the research was conducted in the absence of any commercial or financial relationships that could be construed as a potential conflict of interest.

## Appendix

### Email Texts Used in Experiment 1 and Experiment 2

A. Email texts used in Experiment 1. Brackets illustrate different experimental conditions.

Hello,

I would like to apply for the research assistant position.

I am a foreign graduate student majoring in Psychology.

I am 24 years old and I have been living in the Netherlands for 2 years.

I worked in the past as a research assistant and coordinated a large international project.

I am confident that I can meet the expectations required for the position.

Is this position still relevant? (???)

How soon does the project begin? (???)

Thank you in advance,

Robert (Roberta)

B. Email texts used in Experiment 2. Brackets illustrate different experimental conditions. The texts were originally in Hebrew, translated freely for illustration purposes.

Email 1:

Hi XXX

I would like to ask for your help with defining my email environment very urgently (!!!).

Thank you very much.

Email 2:

Hi XXX

I asked XXX a hundred times already (!!).

I will ask him again. He needs to know that they will not receive their answers in time (!!!).

By the way, when will this be done? (???).

Email 3:

Where is the pickup from? (?!?).

And why did you mention a name of an employee in the list of additional passengers – do I need to pick him up from his home? (???).
