# Supplementary material for: Do You Get What I Mean?!? The Undesirable Outcomes of (Ab)Using Paralinguistic Cues in Computer-Mediated Communication
Source: Front Psychol. 2021 May 12;12:658844. doi: 10.3389/fpsyg.2021.658844 (PMC8149782; doi:10.3389/fpsyg.2021.658844)
Supplement: Supplementary file 1 [file Data_Sheet_1.PDF]

**Supplementary materials: Sample questions from Experiment 1**

**The background scenario:**
We are currently looking for a research assistant to work on an international project.
This project involves interacting with researchers around the globe and collecting data from various locations.
This is a large scale project with important implications
We received many applications and will appreciate your help with sorting them.
Please read carefully the application that was randomly chosen out of the applications we have

The email stimulus (4 conditions – see appendix in manuscript)

Please rate the applicant based on your impressions from the email they sent. Do you think that the applicant is:

| | Not at all | | | Somewhat | | | To a great extent |
|---|---|---|---|---|---|---|---|
| Nice | ○ | ○ | ○ | ○ | ○ | ○ | ○ |
| Positive | ○ | ○ | ○ | ○ | ○ | ○ | ○ |
| Competent | ○ | ○ | ○ | ○ | ○ | ○ | ○ |
| Intelligent | ○ | ○ | ○ | ○ | ○ | ○ | ○ |
| Friendly | ○ | ○ | ○ | ○ | ○ | ○ | ○ |
| Professional | ○ | ○ | ○ | ○ | ○ | ○ | ○ |

To what extent do you think that:

| | Not at all | | | Somewhat | | | To a great extent |
|---|---|---|---|---|---|---|---|
| The applicant is interested in the position | ○ | ○ | ○ | ○ | ○ | ○ | ○ |
| This position is important to the applicant | ○ | ○ | ○ | ○ | ○ | ○ | ○ |
| The applicant wishes to fill the position | ○ | ○ | ○ | ○ | ○ | ○ | ○ |
| | ○ | ○ | ○ | ○ | ○ | ○ | ○ |

To what extent do you recommend the participant for the research assistant position?

| | Not at all | | | Somewhat | | | To a great extent |
|---|---|---|---|---|---|---|---|
| I recommend this applicant | ○ | ○ | ○ | ○ | ○ | ○ | ○ |
| I think that this applicant fits this position | ○ | ○ | ○ | ○ | ○ | ○ | ○ |
| The applicant has the needed skills for this position | ○ | ○ | ○ | ○ | ○ | ○ | ○ |
| This applicant will probably do a very good job on this task | ○ | ○ | ○ | ○ | ○ | ○ | ○ |
